# FascIOtomy: Ultrasound Evaluation of an Intraosseous Needle Causing Compartment Syndrome

**DOI:** 10.5811/cpcem.2018.8.38854

**Published:** 2018-09-05

**Authors:** Tiffany M. Abramson, Laith Alreshaid, Tarina Kang, Thomas Mailhot, Talib Omer

**Affiliations:** Keck School of Medicine of the University of Southern California, LAC+USC Medical Center, Department of Emergency Medicine, Los Angeles, California

## Abstract

Intraosseous (IO) needles are used in critically ill patients when it is not possible to quickly obtain venous access. While they allow for immediate access, IO infusions are associated with complications including fractures, infections, and compartment syndrome. We present a case where point-of-care ultrasound was used to quickly identify a malfunctioning IO needle that resulted in compartment syndrome of the lower extremity.

## INTRODUCTION

Intraosseous (IO) needles are often used in high-acuity patients for whom having vascular access is essential. Malfunctioning IO needles may be associated with patient harm as there is decreased medication delivery and complications secondary to the IO placement, such as compartment syndrome. Further, identifying compartment syndrome may be difficult in critically ill patients who are unable to communicate and for whom serial compartment checks may not be possible or a priority due to competing medical priorities. For this reason, rapid identification of IO needle functionality is needed and can be achieved using point-of-care ultrasound (POCUS). POCUS use has been described as a tool to facilitate evaluation of an IO needle by looking for Doppler signals in the area surrounding the IO during infusions. POCUS can add to patient care and safety by identifying malfunctioning IO needles and potentially preventing complications. We present a case report in which POCUS aided in rapid recognition of IO needle malfunction and in the identification of compartment syndrome.

## CASE REPORT

**Image 1:**
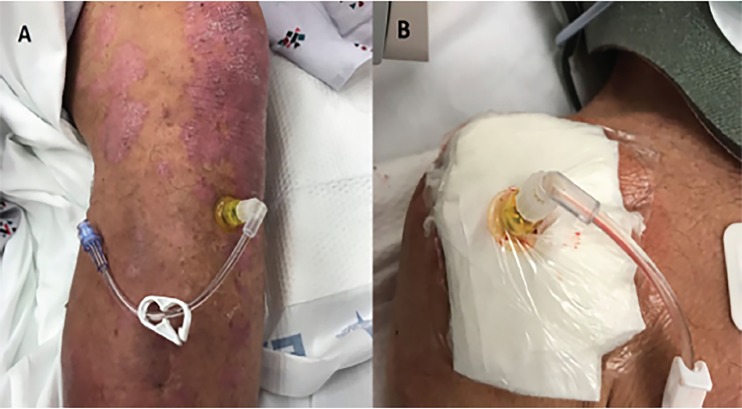
**A)** Photograph of 45 mm intraosseous needle placed to left proximal tibia. **B)** Photograph of 45 mm intraosseous needle inserted to right proximal humerus.

A 63-year-old male was brought into the emergency department (ED) after being found unresponsive. Paramedics in the field noted that the patient was obtunded, with a Glasgow Coma Score of 3 and a blood sugar of 33 milligrams per deciliter (mg/dL). After multiple unsuccessful attempts were made to gain intravenous (IV) access, paramedics used the EZ-IO® to place a 45 mm IO needle into the left proximal tibia(Image 1A)and administered dextrose at a concentration of 50% (D50). Upon arrival to the ED, the patient remained hypoglycemic and unresponsive. He was intubated, and two 50 mL doses of D50 were administered through the IO needle. Nurses noted resistance upon subsequent administration of medications.

**Image 2:**
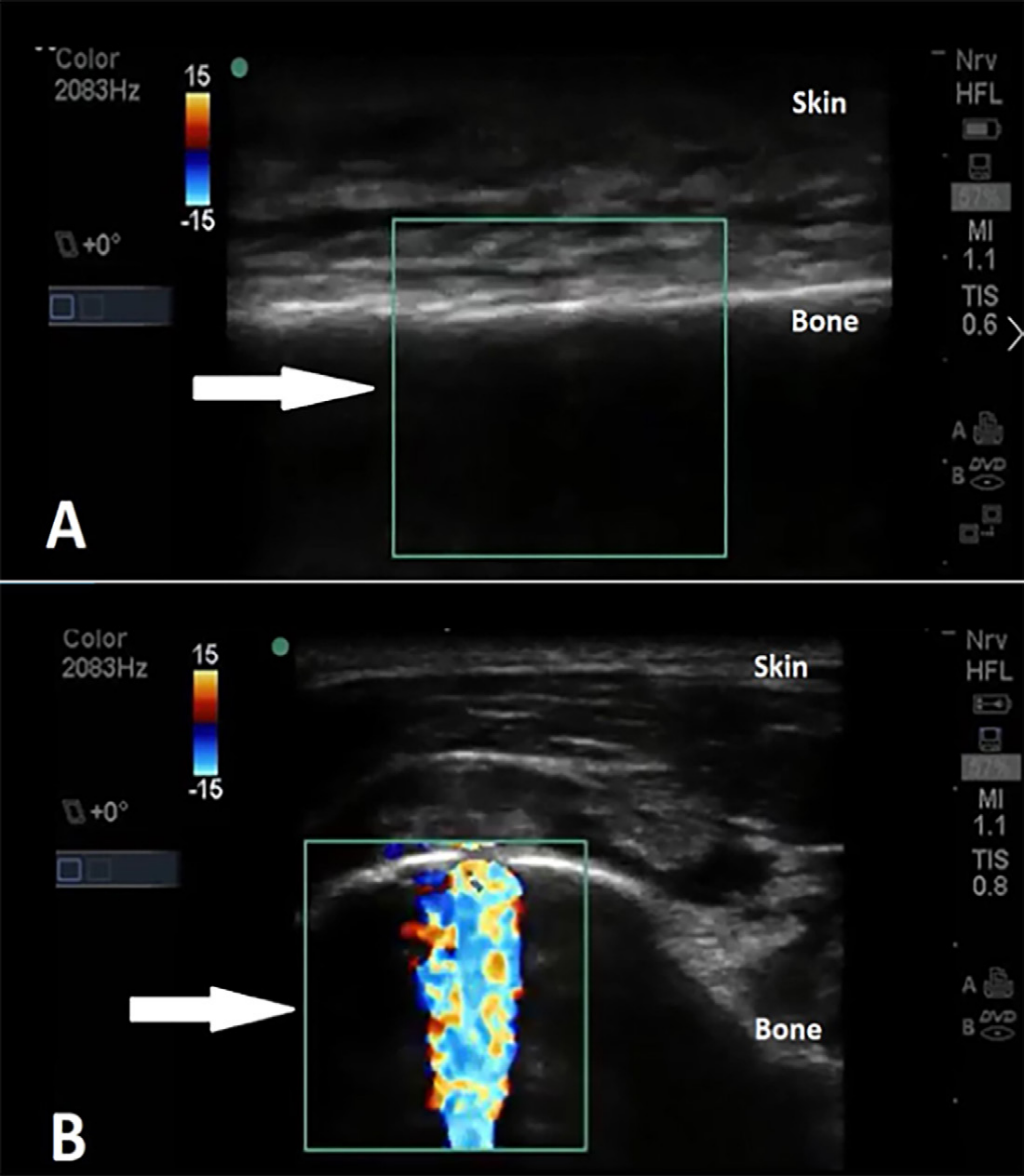
**A)** Ultrasound image of non-functioning left proximal tibia intraosseous needle demonstrating no flow (arrow). **B)** Ultrasound image of properly functioning right humerus intraosseous needle demonstrating flow (arrow).

POCUS was performed to evaluate the functionality of the IO needle. A high-frequency linear probe (5–10 MHz; SonoSite® M-turbo) with color Doppler was used to evaluate the area proximal and distal to the IO access in transverse (short) plane of the tibial bone. Color Doppler showed absence of flow in the IO space during injection of a small amount of normal saline, concerning for inappropriate IO needle placement (Image 2). Subsequently, the tibial IO needle was removed and a second, 45 mm IO needle was placed into the right humerus (Image 1B). The patient was resuscitated and stabilized, receiving medications without complication through the humeral IO infusion.

**Image 3:**
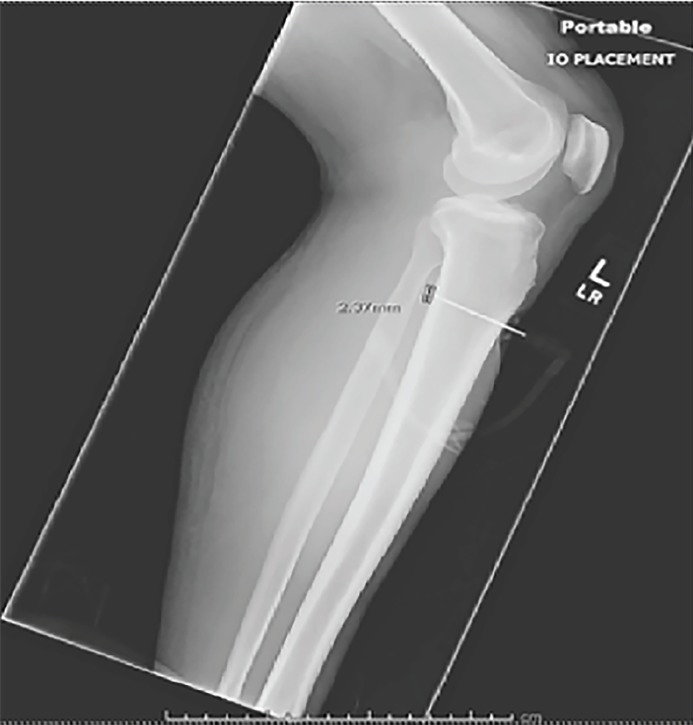
Radiographic demonstrating intraosseous needle extending 2 mm past posterior cortex of tibia.

Fifteen minutes after arrival to the ED, the patient’s left lower extremity was noted to be cool and mottled. Examination of the extremity showed firm compartments and decreased peripheral pulses concerning for compartment syndrome. The deep posterior compartment pressure was 85 mmHg. A radiograph showed that the IO needle had punctured both the anterior and posterior cortex of the tibia, extending 2 mm beyond the posterior cortex (Image 3). The patient was emergently taken to the operating room (OR) by orthopedic surgery for a lower extremity, four-compartment fasciotomy. The procedure demonstrated bulging muscle in all compartments without necrosis. In the OR, all compartments successfully underwent decompression with subsequent return of 2+ palpable distal pulses.

**CPC-EM Capsule**

What do we already know about this clinical entity?

Intraosseous (IO) needle functionality can be assessed using point-of-care color Doppler ultrasound to evaluate for flow in the IO area during infusion through the IO needle.

What makes this presentation of disease reportable?

This is the first reported case in which ultrasound has helped to identify a malfunctioning IO needle and aided in the rapid recognition of a compartment syndrome.

What is the major learning point?

Just as radiographs are used to confirm proper placement of central venous catheters, ultrasound can be used to evaluate placement and functionality of IO needles.

How might this improve emergency medicine practice?

Point-of-care ultrasound can be used to improve patient safety by rapidly identifying malfunctioning IO needles and preventing complications such as compartment syndrome.

## DISCUSSION

IO needles are indicated in critically ill patients for whom peripheral venous access is not possible. Correct placement of the IO needle with the appropriate size needle are both essential to ensure proper function. IO needles may be placed in any large bone with palpable landmarks – usually the distal tibia, proximal tibia, distal femur, or proximal humerus. A 15-mm long needle (pink) is recommended for children (3 kg–39 kg), whereas a 25-mm long needle (blue) is recommended in adults. A 45-mm long needle (yellow) is considered the ideal length for humeral placement in patients with excessive soft tissue or musculature overlying the insertion site.

IO access is considered a fast and effective alternative to peripheral and central lines in critically ill patients. This case, however, showcases a potential risk and complication of its use as well as the value of POCUS in mitigating these risks. The most common complications of IO needle insertion include insertion site infection, hematoma formation, extravasation of medications, and compartment syndrome.^1^,^2^ The incidence of extravasation of IV medications administered through the IO needle is 3.7%.^3^ Compartment syndrome is rare, occurring in 0.6% of cases.^3^–^5^ Nonetheless, compartment syndrome is considered a surgical emergency; thus, prompt recognition of an infiltrated or mispositioned IO needle can avoid this complication and prevent the morbidity associated with neurovascular compromise of the affected limb.^6^,^7^

POCUS is an adjunct that can be used to evaluate functionality of IO needles and ensure safety and efficacy of any medications that may be administered through this access site.^8^ To assess the functionality of the IO needle, a high-frequency linear transducer is placed near the IO site, usually in a transverse plane, and the sample box is manipulated to include the IO space and ideally include the IO needle and surrounding tissue posteriorly. A normal saline flush is then pushed quickly through the IO needle while obtaining real-time imaging focusing on the IO space. Color Doppler signal in the IO space signifies a functioning IO needle. In contrast, absence of signal within the IO space, or signal in the extraosseous compartments including the subcutaneous tissue or muscle signifies extravasation.

## CONCLUSION

Intraosseous needles are often placed in critically ill patients when lifesaving medications need to be administered emergently. A non-functioning or mispositioned IO needle can lead to inadequate medication administration as well as complications, as in our case. It is important to frequently check for signs of infiltration of medications from the IO needle as well as to re-evaluate the neurovascular status of the patient’s limb. In patients with altered mental status in whom a thorough neurovascular exam is difficult, or in an obese patient whose landmarks and compartments may be hard to evaluate, the clinical signs of compartment syndrome can be difficult to assess. By using POCUS to evaluate functionality of the IO needle immediately after placement, clinicians can rapidly confirm properly placed and functioning IO devices, ensuring adequate medication delivery in critically ill patients and potentially avoiding serious complications.

Documented patient informed consent and/or Institutional Review Board approval has been obtained and filed for publication of this case report.

## References

[1] Simmons CM, Johnson NE, Perkin RM (1994). Intraosseous extravasation complication reports. Ann Emerg Med.

[2] Greenstein YY, Koenig SJ, Mayo PH (2016). A serious adult intraosseous catheter complication and review of the literature. Crit Care Med.

[3] Hallas P, Brabrand M, Folkestad L (2013). Complication with intraosseous access: Scandinavian users’ experience. West J Emerg Med.

[4] Al-Ayed T (2008). Gangrene of the leg following Intraosseous Infusion. Ann Saudi Med.

[5] Suominen PK, Nurmi E, Lauerma K (2015). Intraosseous access in neonates and infants: risk of severe complications - a case report. Acta Anaesthesiol Scand.

[6] Gayle M, Kissoon N (1994). A case of compartment syndrome following intraosseous infusions. Pediatr Emerg Care.

[7] Atanda A, Statter MB (2008). Compartment syndrome of the leg after intraosseous infusion: guidelines for prevention, early detection, and treatment. Am J Orthop (Belle Mead NJ).

[8] Tsung JW, Blaivas M, Stone MB (2009). Feasibility of point-of-care colour Doppler ultrasound confirmation of intraosseous needle placement during resuscitation. Resuscitation.

